# Connecting Neuroinflammation and Neurodegeneration in Multiple Sclerosis: Are Oligodendrocyte Precursor Cells a Nexus of Disease?

**DOI:** 10.3389/fncel.2021.654284

**Published:** 2021-06-21

**Authors:** Morgan W. Psenicka, Brandon C. Smith, Rachel A. Tinkey, Jessica L. Williams

**Affiliations:** ^1^Department of Neurosciences, Lerner Research Institute, Cleveland Clinic, Cleveland, OH, United States; ^2^Department of Biological, Geological, and Environmental Sciences, Cleveland State University, Cleveland, OH, United States; ^3^School of Biomedical Sciences, Kent State University, Kent, OH, United States; ^4^Brain Health Research Institute, Kent State University, Kent, OH, United States

**Keywords:** multiple sclerosis, neuroinflammation, oligodendrocyte precursor cell, neurodegeneration, glia, animal models, remyelination

## Abstract

The pathology in neurodegenerative diseases is often accompanied by inflammation. It is well-known that many cells within the central nervous system (CNS) also contribute to ongoing neuroinflammation, which can promote neurodegeneration. Multiple sclerosis (MS) is both an inflammatory and neurodegenerative disease in which there is a complex interplay between resident CNS cells to mediate myelin and axonal damage, and this communication network can vary depending on the subtype and chronicity of disease. Oligodendrocytes, the myelinating cell of the CNS, and their precursors, oligodendrocyte precursor cells (OPCs), are often thought of as the targets of autoimmune pathology during MS and in several animal models of MS; however, there is emerging evidence that OPCs actively contribute to inflammation that directly and indirectly contributes to neurodegeneration. Here we discuss several contributors to MS disease progression starting with lesion pathology and murine models amenable to studying particular aspects of disease. We then review how OPCs themselves can play an active role in promoting neuroinflammation and neurodegeneration, and how other resident CNS cells including microglia, astrocytes, and neurons can impact OPC function. Further, we outline the very complex and pleiotropic role(s) of several inflammatory cytokines and other secreted factors classically described as solely deleterious during MS and its animal models, but in fact, have many neuroprotective functions and promote a return to homeostasis, in part via modulation of OPC function. Finally, since MS affects patients from the onset of disease throughout their lifespan, we discuss the impact of aging on OPC function and CNS recovery. It is becoming clear that OPCs are not simply a bystander during MS progression and uncovering the active roles they play during different stages of disease will help uncover potential new avenues for therapeutic intervention.

## Introduction

Multiple sclerosis (MS) is an inflammatory and neurodegenerative disease of the central nervous system (CNS) characterized by loss of myelin, oligodendrocytes, and axons (Chang et al., 2000, 2002). The global prevalence of MS has increased over the last several years and is highest in North America and Europe (Belbasis et al., 2015). MS represents a large personal and socioeconomical burden. The average age of onset is 30 and 25 years after diagnosis, nearly 50% of MS patients will require permanent use of a wheelchair (Dendrou et al., 2015). Most patients (85%) exhibit the relapsing-remitting form of MS (RRMS), in which disease begins with episodes of neurological dysfunction followed by partial or complete remission, and later progresses to secondary progressive MS (SPMS) with fewer remissions and increasing clinical deterioration (Thompson et al., 1991; Compston and Coles, 2008; Hurwitz, 2009). Some patients (15%) have a third subtype of MS, primary progressive (PPMS), and experience unremitting, progressive loss of neurological function from the onset of disease (Confavreux et al., 2000; Miller and Leary, 2007; Hurwitz, 2009; McCarthy and Weinberg, 2015). Neurological disability in RRMS is due to immune-mediated demyelination, while SPMS and PPMS is dominated by neurodegenerative processes (Miller and Leary, 2007). Thus, it is not surprising that the 12 FDA-approved immunomodulatory therapies have demonstrated efficacy for RRMS, while a single approved therapy may be beneficial for SPMS or PPMS, although long-term studies are still required (Noseworthy et al., 2000; Leary and Thompson, 2003; Wingerchuk and Carter, 2014; Feinstein et al., 2015; Montalban et al., 2017). It is also acknowledged that while the standard therapies for treating MS reduce relapses in RRMS patients (Haghikia et al., 2013; Feinstein et al., 2015), they do not halt neurodegeneration and disability continues, becoming permanent. Investigating the potential link between neuroinflammatory and neurodegenerative processes during autoimmunity may uncover a source of novel therapeutic potential for MS patients.

MS is a neurodegenerative and inflammatory disease mediated by autoreactive immune cells that initiate myelin and axon injury in a progressive manner, which leads to sustained motor and sensory function loss (Trapp et al., 1998; Noseworthy et al., 2000; Trapp and Nave, 2008). Following myelin destruction during the pathogenesis of MS, axons are left exposed, inefficient, and susceptible to degeneration. During remyelination, damaged axons reacquire a myelin sheath and recover lost function (Franklin, 2002). Clinically, this process occurs early on and requires the generation of new oligodendrocytes (OLs) (Keirstead and Blakemore, 1997), the myelinating cell of the CNS, from oligodendrocyte precursor cells (OPCs) (Watanabe et al., 2002). Although there is also evidence to suggest that mature OLs too can participate in remyelination of MS lesions (Duncan et al., 2018). Importantly, in the harsh lesion environment, remyelination does occur (Prineas et al., 1993). However, while there are OPCs found in and around MS lesions (Chang et al., 2002), remyelination efficiency wanes with patient age and as lesions and the disease become more chronic (Franklin, 2002; Frischer et al., 2015; Gruchot et al., 2019). OPCs are typically more sensitive to growth factors produced by resident CNS cells compared to mature myelinating OLs, which are largely unresponsive to identical factors, although the molecular mechanisms responsible for this dichotomy remain poorly understood (Moore et al., 2015). Functionally, the maturation of OPCs to OLs is vital to regain homeostasis as this conversion provides critical structural, metabolic, and trophic support to neuronal axons (Funfschilling et al., 2012; Lee et al., 2012; Duncan et al., 2021). However, within the MS lesion environment, there are several factors that can suppress proper OPC function, preventing successful remyelination and, thus, potentially contributing to neurodegeneration and dampening clinical improvement (Compston and Coles, 2002; Franklin, 2002; Gruchot et al., 2019).

The extracellular milieu in which OPCs reside can heavily influence their ability to migrate, proliferate, and/or mature into myelinating OLs. Within the MS lesion, there are a variety of both resident CNS cells and infiltrating peripheral immune cells, although the type and amount of each cell population varies depending on the lesion type (Bo et al., 1994; Bruck et al., 1994, 1995; Trapp et al., 1998). While the etiology of MS is largely unknown, it is well-established that subsets of infiltrating T cells heavily contribute to pathology with the help of peripheral monocytes, macrophages, and the CNS resident innate immune cells, microglia. While there are multiple routes of entry, a primary method of gaining access to the CNS by peripheral leukocytes is to breach the endothelial (and pericyte) layer of the blood-brain barrier (BBB). There, they are able to then penetrate the glia limitans and the astrocyte endfeet of the neurovascular unit, responding to localizing cues and upregulating adhesion molecules via the release of inflammatory mediators (Lopes Pinheiro et al., 2016; Prinz and Priller, 2017; Williams et al., 2020). In addition to this intricate, pathological interplay among cells of the periphery and CNS, there is significant heterogeneity among the cell populations that contribute to this complex neuroinflammatory lesion environment. While several T cell subsets are known to perpetuate inflammation in MS and in many experimental models of MS, particularly in acute phases of disease, mice that lack CD4^+^ and CD8^+^ T cells have significant deficits in remyelination (Bieber et al., 2003), highlighting the complicated nature of neuro-immune interactions ongoing in MS. Beyond the complexities of peripheral immune cell contributions to pathology and repair, microglia and astrocytes exhibit an exceptional amount of genetic, morphological, and functional heterogeneity depending on microenvironmental cues (Masuda et al., 2020; Escartin et al., 2021), which can significantly influence how OPCs function as glia are highly communicative with other glia (Peferoen et al., 2014; Liu et al., 2020; Nutma et al., 2020) as well as with neurons (Stogsdill and Eroglu, 2017). Here, we will discuss the underlying pathological neuroinflammatory processes and factors that contribute to neurodegeneration and MS progression, focusing on the role of glial intercellular communication, the microenvironment, and OPCs.

## Multiple Sclerosis Lesions

Between 1862 and 1865, colleagues Jean Martin Charcot and Alfred Vulpian identified a form of spinal MS. Then, in April of 1866, Charcot macroscopically described nearly identical sclerotic lesions in both the brain and spinal cord during an autopsy. He later presented microscopic characteristics of these lesions, identifying a sharp boarder between the lesion and normal-appearing tissue with a transition zone, and a lesion center consisting of “greasy droplets” that resembled myelin and a description of axonal degeneration (Zalc, 2018). In more recent years, the characterization of MS lesions has been extensively described (Lassmann et al., 1998; Trapp et al., 1998, 2018; Kuhlmann et al., 2017), although new knowledge regarding the lesion environment is continually being uncovered. While lesions are found in both white and gray matter, they are predominantly localized to white matter tracts and are typically categorized into active, chronic active, and inactive, with lesions often transitioning between types (Trapp et al., 1998; Kuhlmann et al., 2017). Myelocortical lesions were also recently described in the gray matter of a subset of MS patients and may indicate that neuron degeneration can occur independently of cerebral white matter demyelination (Trapp and Ontaneda, 2018). Understanding the pathophysiology underlying these various lesion types may hint at the potential link between neuroinflammation and neurodegeneration and uncover possible interventions to prevent permanent disability in MS patients.

Active lesions are characterized as having a significant amount of peripheral immune cell infiltration, often including a substantial population of CD8^+^ T cells, CD20^+^ B cells, and macrophages, along with some CD4^+^ T cells (Correale and Villa, 2010; Disanto et al., 2012; Machado-Santos et al., 2018). These lesions also have involvement from CNS resident cells including reactive astrocytes and activated microglia throughout the lesion, with a high concentration of microglia ringing the lesion edge (Frischer et al., 2015). Active lesions are highly inflammatory, largely due to the quantity of infiltrating cells producing a myriad of cytokines and other inflammatory factors (Cannella and Raine, 1995; Kutzelnigg et al., 2005; Frischer et al., 2009), which contribute to demyelination and eventual axonal loss (Trapp et al., 1998). However, despite the inflammatory nature of active lesions, oligodendrocytes often reappear during early stages of remyelination in acute MS, contributing to partial or complete recovery (Lassmann, 1983; Prineas et al., 1993; Raine and Wu, 1993; Lucchinetti et al., 1999, 2000; Barkhof et al., 2003; Patrikios et al., 2006). RRMS is typically associated with predominately active lesions and recovering/remyelinating plaques (Harris et al., 1991; Thompson et al., 1991). In patients with RRMS, axon signal conduction tends to be reduced within active lesions, which can lead to a very heterogenous profile of symptoms, depending on the CNS region in which the lesion is located (Kutzelnigg et al., 2005; Campbell et al., 2012; Heß et al., 2020). During RRMS and in early SPMS and PPMS, the majority of lesions are active and chronic active. However, in more chronic stages of MS, the proportion of lesions begin to shift more toward inactive (Luchetti et al., 2018).

Chronic active lesions are often referred to as slowly expanding or smoldering lesions and are characterized by fewer infiltrating cells, further accumulation of microglia on lesion edges, with fewer microglia located in the center of the lesion compared to active lesions (Dal-Bianco et al., 2017; Absinta et al., 2018; Calvi et al., 2020). While demyelination still occurs in chronic active lesions, particularly along the lesion edge, there is a significant reduction in the rate of demyelination. This is accompanied by a reduction in remyelination relative to active lesions (Absinta et al., 2019; Wang et al., 2019; Elliott et al., 2020), with the center of the lesion remaining demyelinated and unlikely to remyelinate (Calvi et al., 2020; Elliott et al., 2020; Heß et al., 2020). In the center of chronic active lesions, reactive astrocytes proliferate and form a dense network of overlapping astrocytes with few axons, referred to as the glial scar (Voskuhl et al., 2009; Sofroniew and Vinters, 2010; Jonkman et al., 2015; Kuhlmann et al., 2017). In a recent *in vivo* cohort study, chronic active lesions were detected by magnetic resonance imaging using their characteristic rims and were associated with an aggressive disease course and poorer clinical outcomes (Absinta et al., 2019). Inactive lesions are associated with prolonged disease duration, and unlike active and chronic active lesions, are characterized by a lack of infiltrating cells and microglia, as well as a stark loss of axons and near complete depletion of mature oligodendrocytes, leading to their static nature (Brück et al., 1996; Correale and Villa, 2010; Jonkman et al., 2015; Kuhlmann et al., 2017). However, analogous to chronic active lesions, gliotic scars consisting of astrocytes are still present (Kornek et al., 2000; Peterson et al., 2001).

Active, chronic active, and inactive lesions occur simultaneously in MS patients but tend to shift in predominance depending on disease chronicity (Brück et al., 1996; Kornek et al., 2000; Frischer et al., 2015; Jonkman et al., 2015; Kuhlmann et al., 2017; Heß et al., 2020). Lesions tend to progress from active to chronic active to inactive as an individual lesion progresses, with some exceptions (Harris et al., 1991; Thompson et al., 1991; Kuhlmann et al., 2017; Absinta et al., 2018). The most notable exceptions are active lesions, which can completely or partially remyelinate rather than progressing onto chronic active depending on lesion severity (Frischer et al., 2015; Jonkman et al., 2015). Additionally, inactive and later stage chronic active lesions can be reactivated to a more active lesion phenotype (Thompson et al., 1991; Kuhlmann et al., 2017; Absinta et al., 2018), suggesting that inactive lesions may be reinfiltrated by a new wave of macrophages/microglia. Interestingly, macrophages occupying active and chronic active lesions often contain lipids, presumably from ingesting myelin debris, and are immunomodulatory rather than inflammatory in nature (Boven et al., 2006). This heterogenous mix of lesion types, and thus cellular composition, may partially account for the inefficacy of currently approved immunomodulatory drugs in treating SPMS and PPMS patients, as the role of inflammation can vastly differ between lesion types (Bates, 2011; Ciotti and Cross, 2018). Limiting inflammation in active lesions is certainly beneficial; however, in lesions lacking significant inflammation, the impact of immune modulation is likely minimal, or may even have potentially negative consequences, in chronic active and inactive lesions. To this point, remyelination most frequently occurs around the borders of lesions where inflammation is most pronounced (Brück et al., 1996; Jonkman et al., 2015; Wang et al., 2019; Calvi et al., 2020); and, many animal studies have revealed a critical role for immune components in CNS remyelination and repair, particularly during chronic stages of disease. To better understand the complex nature of the CNS environment during MS, several murine models have been developed and are critical in uncovering novel therapeutic modalities and elucidating multiple aspects of disease.

## Murine Models of MS

Progressively sophisticated animal models of human neurological pathology are continuously being developed and improved. These models make it feasible to examine complex cellular interactions within the CNS and their contributions to neuroinflammation and disease progression (Kipp et al., 2017). While no animal model perfectly recapitulates observed MS pathology, there are components of any given model which are useful, replicating different clinical, immunological, and histological aspects of human disease. Genetic manipulations using the Cre/*lox* and other systems in combination with some murine models of MS are valuable tools in elucidating specific molecular pathways important for MS pathology and repair.

### Experimental Autoimmune Encephalomyelitis (EAE)

EAE is the most commonly used animal model for MS, sharing many features of the human disease, with the inflammatory components being of particular interest (Borjini et al., 2016). The classical EAE phenotype, characterized by ascending flaccid paralysis and preferential immune infiltration of the spinal cord, has been widely utilized since its characterization in the 1930s (Croxford et al., 2011). Ascending paralysis correlates with peripheral immune infiltration and inflammation in the lumbar region of the spinal cord, which becomes progressively more inflamed throughout the acute phase of disease (Miller et al., 2010). Clinical signs, most notably, an abnormal gait, tail paralysis, and hindlimb paralysis, are scored using a standard system. In general, the EAE model yields high disease incidence with a consistent and robust disease course that is very replicable (Stromnes and Goverman, 2006), and though not fully understood, is nonetheless well-characterized. Shared aspects of EAE and MS pathology include the targeted destruction of myelin accompanied by axonal degradation, which results in multiple disseminated lesions, predominantly located in perivascular spaces (Kornek et al., 2000). Likewise, both EAE and MS share common temporal characteristics, evidenced by inflammatory lesion development, followed by demyelination, gliosis, decreased frequency of lesion-associated mononuclear cell infiltrates, and limited remyelination (Storch et al., 1998). However, EAE is induced via external immunization while MS occurs spontaneously (Stromnes and Goverman, 2006). As such, EAE can represent many inflammatory characteristics of MS including peripheral immune cell priming, followed by CNS infiltration, and subsequent CNS resident cell responses (Baxter, 2007). Model heterogeneity exists within EAE, with various antigenic targets and murine strains being used. The most common immunizing antigens include myelin-associated glycoprotein, myelin basic protein, oligodendrocytic basic protein, myelin oligodendrocyte glycoprotein (MOG), and proteolipid protein (PLP). The target of choice is dependent on the genetic background of the strain being used. EAE is most commonly induced in rodents, which have an easily and consistently replicable disease induction, with mice used preferentially over rats in the last few decades (Miller et al., 2010; Croxford et al., 2011).

EAE is primarily considered a CD4^+^ T cell-mediated disease (Van Kaer et al., 2019), although the particular role of various CD4^+^ T helper cell subsets and, to an extent, CD8^+^ cytotoxic T cells are also well-appreciated (Sun et al., 2001; Sonobe et al., 2007). However, like MS, EAE has a complex immune profile in which natural killer T cells (Jahng et al., 2001; Singh et al., 2001; Denney et al., 2012), γδT cells (Blink and Miller, 2009), mucosal invariant cells (Pál et al., 2001; Mars et al., 2002; Croxford et al., 2006), and B cells (Matsushita et al., 2008; Pöllinger et al., 2009; Pierson et al., 2014; Harp et al., 2015) each contribute to pathology. Further, other adaptive and innate leukocytes can have differing roles, imposing either regulatory or pathogenic functions depending on the disease state, which have alternate contributions to EAE pathogenesis (Mcginley et al., 2018; Van Kaer et al., 2019).

There are two primary methods commonly used to induce EAE: active immunization using a myelin antigen with adjuvants (typically complete Freund's adjuvant and pertussis toxin) and passive induction via adoptive transfer of activated, myelin-specific T cells from an immune-primed donor into a naïve recipient without the use of adjuvants (Stromnes and Goverman, 2006). Actively induced EAE in the C57Bl/6 mouse strain is typically a biphasic model, with an induction phase and an effector phase (Miller et al., 2010). During induction, similar to early lesions in RRMS, focal vascular disruptions and increased BBB permeability precede microglial activation and peripheral leukocyte infiltration (Alvarez et al., 2015; Barkauskas et al., 2015). Next, myelin-specific T cells extravasate across the endothelial layer and into the CNS and initiate pro-inflammatory signaling cascades to promote the recruitment of B cells (Furtado et al., 2008; Matsushita et al., 2008), innate immune cell infiltration, and T cell reactivation (Pierson et al., 2014). These primed T helper cells secrete a number of cytokines, including interferon (IFN)γ and tumor necrosis factor (TNF)α, which activate microglia and peripheral macrophages (Ponomarev et al., 2005; Ajami et al., 2011). Of note, OPCs are also responsive to (Rodgers et al., 2015) and secrete cytokines (Moore et al., 2015) and accumulate perivascularly, contributing to BBB compromise during EAE (Girolamo et al., 2019). Further, this inflammatory cytokine cascade leads to increased phagocytic activity by activated mononuclear cells and enhanced cytotoxic effects of cytokines secreted by CD4^+^ T cells and monocytes, enhancing the demyelination of axons, eventual axonal transection, and neuronal cell death (Miller et al., 2010). Even as the acute inflammatory response fades and levels of cellular infiltrates decreases, axons continue to degenerate. In C57Bl/6J mice immunized with the myelin peptide MOG_35−55_, axonal density was reduced within lesions at early and chronic time points and significant axonal loss was observed in both the gray matter and normal-appearing white matter, which was associated with clinical impairment (Herrero-Herranz et al., 2008). Similarly, axonal pathology is a hallmark of MS and a substantial contributor to irreversible clinical disability (Trapp et al., 1998). Passive EAE via adoptive transfer of activated, myelin-specific T cells into naïve hosts can be used to study the effector phase without introducing potentially confounding effects of the adjuvants used for active immunization (Brocke et al., 1996; Stromnes and Goverman, 2006). Additionally, compared to active EAE, passive EAE is highly synchronous and consistent. Although a limitation of passive EAE is limited microglial activation, the absence of sustained demyelination and variable axonal injury, it is extremely useful for the study of immune control mechanisms, T cell-mediated neuroinflammation induction, and immune cell-specific mechanisms of tissue injury (Kipp et al., 2012; Lassmann and Bradl, 2017).

There are several variations of EAE aside from the C57Bl/6 model used to study various aspects of MS pathology. Other commonly used models include SJL/J mice immunized against the myelin peptide PLP_139−151_ (Tuohy et al., 1989). This model has a number of similarities with the C57Bl/6 EAE model in terms of pathology; however, an advantage to the SJL/J model is that mice develop a progressive relapsing-remitting disease course (Whitham et al., 1991; Miller and Karpus, 2007). Relapsing-remitting EAE can be induced via active immunization or by transfer of activated PLP-specific T cells, the latter of which is associated with epitope spreading (McRae et al., 1995) or the development of immunity to secondary endogenous antigens following initial immune priming to a self-antigen (Vanderlugt and Miller, 1996). While this model suffers from the lack of genetic deletion strategies, it is useful for testing novel treatment strategies with respect to additional clinical parameters including reductions in relapse rate. Finally, some models of EAE yield an alternative clinical presentation, referred to as atypical EAE, which often display ataxia, head tilting, and axial rotation associated with parenchymal hindbrain inflammation (Simmons et al., 2013). This is most often seen in animals with a disruption in IFNγ signaling (Wensky et al., 2005; Lees et al., 2008; Liu et al., 2015). Some models present with a mixed phenotype developing both atypical and classical signs (Pierson et al., 2012), mimicking the heterogenous regional localization of MS lesions in patients.

### Viral Encephalomyelitis

Several viruses initiate chronic infections in the murine CNS, serving as useful models for the study of axonal damage and inflammatory-induced demyelination. Two well-characterized demyelinating viral models involve encephalomyelitis induced by the RNA viruses Theiler's murine encephalomyelitis virus (TMEV), a member of the non-enveloped *Picornaviridae* family, and mouse hepatitis virus (MHV), a member of the enveloped *Coronaviridae* family (Miller et al., 2001; Stohlman and Hinton, 2001; Bergmann et al., 2006; Mecha et al., 2013).

#### TMEV

Similar to EAE, the pathology of TMEV-induced encephalomyelitis is dependent on the dose administered, the viral strain, and the genetic background of the mouse. There are highly neurovirulent strains, such as GDVII and FA, which induce an acute, often fatal encephalitis (DePaula-Silva et al., 2017; Savarin and Bergmann, 2017; Libbey and Fujinami, 2021). There are also attenuated variants of TMEV, including BeAn and the Daniel's strain (DA). Infecting the CNS of susceptible SJL/J mice with an attenuated strain generates a biphasic CNS pathology, while infection of C57Bl/6J induces an acute infection, which is rapidly cleared (Zoecklein et al., 2003; Richards et al., 2011). Therefore, TMEV intracerebral infection of SJL/J mice is the most commonly used MS animal model as it produces acute encephalomyelitis followed by a chronic demyelinating phase, primarily in the spinal cord. The chronic demyelinating stage is called TMEV-induced demyelinating disease (TMEV-IDD) (Bröer et al., 2016). Unlike other models, TMEV-IDD lacks substantial remyelination due to its progressive nature, so it does not easily mirror a relapsing-remitting phenotype (Ulrich et al., 2008). Thus, TMEV most effectively models MS types with hallmark chronic, progressive immune-mediated demyelination with remyelination failure as TMEV-IDD animals show a continuous increase in motor disability which coincides with increases in white matter damage (Sun et al., 2015; Leitzen et al., 2019). The number of OPCs within TMEV-IDD lesions is transiently higher than homeostatic levels. GFAP^+^ cells are likewise present in elevated numbers in spinal cord lesions. Interestingly, lesion-associated NG2^+^ OPCs can co-express GFAP or CNPase suggesting potential alternative routes of OPC differentiation, which may affect remyelination capacity (Ulrich et al., 2008). Both the BeAn and DA strains can induce TMEV-IDD, but there are strain-related differences in pathology. BeAn infection in susceptible SJL/J mice develop clinical signs similar to those observed in EAE, including irregular gait and hind limb paralysis. DA-infected mice develop the same clinical features, but not until 140–180 days post-infection (Oleszak et al., 2004).

Following the acute phase of TMEV-DA infection, viral replication persists at low levels in macrophages and microglia (Clatch et al., 1990; Qi and Dal Canto, 1996; Jelachich and Lipton, 1999) and to a lesser extent in astrocytes and OLs (Zheng et al., 2001). There is immune infiltration in the hindbrain subcortical gray matter but little to no white matter infiltration throughout the acute phase (Oleszak et al., 2004). During the chronic phase, depending on viral dosage and host age, susceptible SJL/J mice develop MS-like chronic neuroinflammation which presents as functional motor deficits. TMEV-DA-induced lesions mimic features of MS cortical lesions in that chronic demyelinating white matter has extensive peripheral leukocyte infiltrates, the majority of which are CD4^+^ (Palma et al., 1999; Mohindru et al., 2006) and CD8^+^ T cells (Begolka et al., 2001; Lyman et al., 2004), though some monocytes/macrophages (Mack et al., 2003) and comparably few B cells are also present (Gilli et al., 2015). In particular, PLP-reactive CD4^+^ Th1 cells have been implicated in the perpetuation of disease (Katz-Levy et al., 2000) and epitope spreading (Miller et al., 1997; Mcmahon et al., 2005) has been demonstrated to strengthen myelin antigenic responses at 2–4 weeks (McCarthy et al., 2012; DePaula-Silva et al., 2017). This leads to extensive white matter damage and axonal loss in TMEV-infected mice, particularly in the thoracic and cervical spinal cord, as early as 7 days post-infection (Leitzen et al., 2019). Over the following weeks, damaged axons are surrounded by locally activated macrophages/microglia without peripheral T cell recruitment or demyelination, which does not occur until the chronic phase. Interestingly, areas of axonal damage corresponded to chronic demyelination late in infection (Tsunoda and Fujinami, 2010), which may mirror aspects of MS where neuronal death precedes demyelination and T cell involvement.

#### MHV

Studies employing MHV-induced demyelination have largely focused on two neurotropic strains: the mildly neurovirulent and hepatotropic MHV-A59 strain and a neuroattenuated variant of the lethal JHM strain, designated 2.2-V-1 (Fleming et al., 1986; Bergmann et al., 2006; Templeton et al., 2008; Bender and Weiss, 2010; Bender et al., 2010). Following intracerebral inoculation, CNS cells become acutely infected. After infection, chronic demyelination occurs. Clinical signs such as hind limb paralysis correlate with neuroinflammation and white matter damage (Savarin and Bergmann, 2017). The virus infects and replicates within ependymocytes, microglia, astrocytes, and OLs, comparatively sparing neurons (Wang et al., 1992). Control of virus in astrocytes and microglia/macrophages is perforin-mediated (Lin et al., 1997; Bergmann et al., 2004), while IFNγ controls viral replication within OLs (Parra et al., 1999; Bergmann et al., 2004). The MHV-A59 strain infects neurons and glial cells and induces a mild encephalitis (Lavi et al., 1999; Phillips et al., 2002), whereas the JHM v2.2-1 variant is more gliatropic (Lin et al., 1997; Bergmann et al., 2004), largely sparing neurons, and causes a more severe encephalitis which progresses to hind limb paralysis from which the majority of mice recover (Rempel et al., 2004; Bender and Weiss, 2010). Despite the distinct cellular tropisms, both virus strains spread from the brain to the spinal cord and infectious virus is generally controlled within 10–14 days. Of note, viral RNA persists in spinal cords for several months, up to a year post-infection in the case of MHV-JHM v2.2-1 (Savarin and Bergmann, 2017). Demyelination is prominent in spinal cord white matter 14 to 30 days post-infection, after initial virus control, depending on the MHV strain and age at infection. A caveat is that many MHV-A59 studies are carried out in 4–5 week-old mice, whereas MHV-JHM v2.2-1 studies administer virus to 6–7 week-old mice preempting direct comparisons (Lavi et al., 1984; Fleming et al., 1987; Jordan et al., 1989; Templeton and Perlman, 2007). Both infections cause the upregulation of numerous chemokines and cytokines to enhance recruitment of various leukocytes including neutrophils, monocytes, natural killer cells, CD4^+^ and CD8^+^ T cells as well as various B cell subsets in a regulated, temporal pattern, yet the magnitude of inflammatory mediators as well as leukocyte subsets is dependent on the virus variant (Williamson et al., 1991; Bergmann et al., 2001; Liu and Lane, 2001; Tschen et al., 2002). CD8^+^ T cells, with the help of CD4^+^ T cells, are the main effector cells in viral control (Phares et al., 2012). Despite a potent T cell mediated response, upregulation of inhibitory ligands, like programmed death-ligand 1, counteract exuberant effector function to limit pathology potentially contributing to persistence (Phares et al., 2010; Puntambekar et al., 2011). Demyelination is immune-mediated as immunodeficient (SCID and RAG-deficient) mice do not develop overt demyelination (Houtman and Fleming, 1996; Wu and Perlman, 1999) and both virus-specific CD4^+^ and CD8^+^ T cells alone can mediate demyelination (Bergmann et al., 2004; Stohlman et al., 2008). Moreover, microglia and macrophages have been shown to mediate demyelination (Savarin et al., 2016). This overt demyelination lends itself to elegant remyelination studies as chemokines like CXCR2 are highly upregulated and are critical to OPC-mediated repair (Marro et al., 2019; Skinner and Lane, 2020). Despite an overall reduction in T cells during viral persistence, new lesions are continuously formed as shown by lipid laden myeloid cells (Wu et al., 2000; Liu et al., 2001; Rempel et al., 2004), consistent with MS lesions.

### Chemical or Toxin-Induced Demyelination Models

Chemical and toxin-mediated demyelination models largely lack the immune component relevant to EAE, TMEV, and MHV. Inflammatory models indeed more closely resemble MS pathology in many ways, but are more complex and are less consistent in terms of demyelination chronology and lesion localization, extent, and characteristics. Inflammation-independent models are nonetheless useful for in-depth analyses of CNS resident cells engaged in the de- and remyelination process and are often employed to investigate therapeutics which limit demyelination or stimulate remyelination (Procaccini et al., 2015). Gliotoxic agents ethidium bromide (EtBr) and lysolecithin induce focal demyelination at their injection site, while the systemically administered copper chelator cuprizone (CPZ) induces reversible white and gray matter demyelination throughout the brain (Blakemore and Franklin, 2008; Bai et al., 2016; Lubetzki et al., 2020).

#### EtBr and Lysolecithin

The EtBr model has seen extensive use in rats and remains preferentially a rat model, though it has recently been used in mice, revealing subtle differences in pathology between the two rodents. EtBr is a DNA intercalator and its toxicity preferentially impacts astrocytes by restricting their ability to release trophic factors leading to associated OLs to die, along with the affected astrocytes, while largely sparing neurons. The death of these glia in and around the injection site results in localized demyelination (Torre-Fuentes et al., 2020). Immunoreactive GFAP^+^ astrocytes are then recruited, but remain confined to the lesion perimeter. EtBr injection into murine ventral spinal cord white matter induces hind limb motor deficits, similar to those seen in other models, which can persist with accompanying CD45^+^ infiltrating immune cells and axonal loss in chronic stages (Kuypers et al., 2013). While EtBr has been used in mice, the studies outlining its use are limited. Lysolecithin is more commonly used and as a result has been more thoroughly characterized.

Lysolecithin is a phospholipase A2 activator, which, much like EtBr, induces a highly reproducible focal demyelination in CNS white matter (Procaccini et al., 2015). The toxin acts as a detergent-like agent directly deteriorating myelin sheaths by solubilizing the lipid membrane, effectively destroying myelin while sparing other cells and cellular components. As a result, OPCs are spared and lysolecithin-induced lesions remyelinate faster than most other demyelination models (Bjelobaba et al., 2018), with nearly all axons encased in myelin sheaths by day 23 post-injection (Jeffery and Blakemore, 1995), offering a consistent, timely, and reproducible method for studying OPCs in the context of de- and remyelination events. Interestingly, while demyelination has been described as being T and B cell-independent, CD4- and CD8-deficient mice had significantly reduced remyelination at the injection site compared to controls, indicating T cells may be involved in myelin repair and proper OPC function (Bieber et al., 2003). Unlike EtBr and CPZ, lysolecithin is not considered to be fully immune-independent, acting as a chemoattractant to monocytes and initiating a limited focal inflammatory response (Torre-Fuentes et al., 2020) and remyelination that is accompanied by significant astrogliosis (Jeffery and Blakemore, 1995). While useful, EtBr and lysolecithin are technically demanding models and de- and remyelination is restricted to the site of injection.

#### Cuprizone

CPZ, a low molecular weight copper chelator, is systemically administered to mice typically by incorporating it into standard chow. Copper is an enzymatic cofactor necessary for many metabolic functions, including ATP production, and while its exact mechanism of action is unknown, it is generally accepted that the chelation of copper is highly disruptive to the robust cellular metabolism of OLs (Praet et al., 2014). Within myelinating OLs, it can cause the formation of cellular megamitochondria, ATP shortages, reactive oxygen species build up, and endoplasmic reticulum stress, all of which OLs are particularly vulnerable to due to their highly intensive metabolism required for myelin production. Additionally, disruption of oxygen scavenging by CPZ can be exacerbated and catalyzed via the Fenton reaction by the high amount of sequestered iron within cells, producing more reactive oxygen species (Praet et al., 2014). T cell suppression occurs during demyelination, which allows the effects of CPZ to be discriminated from particular immune compartments. Demyelination in both the gray and white matter of the brain are highly reproducible, synchronous, and anatomically distinct during the time mice are fed CPZ-embedded chow (Matsushima and Morell, 2001; Torre-Fuentes et al., 2020) unlike the demyelination that is seen in EAE or in virally-induced demyelination. Withdrawal of the CPZ diet also allows consistent remyelination. This feature enables precise timing and elucidation of specific mechanisms, particularly those active in CNS glia, during de- and remyelination. While peripheral immune cell infiltration is limited in this model, CXCR2^+^ neutrophils are thought to be critical for demyelination (Liu et al., 2010). CPZ is therefore ideal for the study of de- and remyelination with a focus on resident CNS cells and perhaps an ideal model for studying a “two-hit” mechanism, relevant in MS pathology.

Like many models for MS, there are differences in CPZ-induced pathology depending on the CPZ dosage and mouse strain used. Using 0.2% CPZ chow in 8–10 week-old C57Bl/6J mice is the most commonly used and well-characterized models. In C57Bl/6 mice, 0.2% CPZ induced reliable and complete demyelination of the caudal corpus callosum and superior cerebellar peduncle without significant weight loss or liver toxicity (Hiremath et al., 1998). The typical timeframe of CPZ exposure is 5–6 weeks, with early demyelination occurring as early as 3 weeks with a significant presence of NG2^+^ OPCs and microglia within the corpus callosum (Hiremath et al., 1998; Mason et al., 2000; Williams et al., 2014). This is accompanied by significant gliosis, largely due to proliferating astrocytes. These astrocytes are thought to contribute to damaged myelin clearance via recruitment of microglia as ablation of GFAP^+^ astrocytes during CPZ intoxication resulted in decreased microglial activation, excess myelin debris, impaired OPC proliferation, and a delay in remyelination (Skripuletz et al., 2013). Microglia and astrocytes also significantly upregulate interleukin (IL)-1β, which is thought to induce the release of insulin-like growth factor (IGF)-1 during CPZ demyelination (Mason et al., 2001; Matsushima and Morell, 2001), suggesting a role for these glia in a return to homeostasis. A clear perk of the CPZ model of demyelination is that remyelination occurs rapidly and spontaneously following the cessation of CPZ treatment with sequential myelin proteins expressed as early as 1 week post-CPZ cessation (Lindner et al., 2008). Importantly, chronic exposure to CPZ (12–16 weeks) results in axonal damage, even with concurrent remyelination. Although, remyelination using this chronic paradigm is delayed by 2-fold compared to mice fed CPZ for 6 weeks (Lindner et al., 2009). Although remyelination occurs in MS lesions, it is often inhibited, particularly in chronic disease stages; thus, this model represents a plausible way to elucidate potential mechanisms of remyelination failure in MS.

Other variations of the CPZ model include combining components of EAE induction to establish MS-like lesions in the brain. In most iterations of EAE, there is little to no immune cell infiltration in the cerebrum; however, immunization of C57Bl/6 mice fed CPZ with MOG_35−55_ resulted in significant lesions, characterized by T cell accumulation and axonal damage in the demyelinated corpus callosum, which were detectable by magnetic resonance imaging (Boretius et al., 2012). Another variation of this combined approach utilizes mice pre-treated with CPZ for 3 weeks, followed by standard chow for 2 weeks to establish focal lesions. Mice were then immunized with the MOG_35−55_ peptide, which induced significant forebrain lesions within white matter tracts and cortical and subcortical gray matter. Further, in addition to demyelination, these lesions exhibited discontinuation of the glia limitans, infiltration of neutrophils and granulocytes, and focal axonal damage (Scheld et al., 2016; Ruther et al., 2017). Combining passive transfer EAE with CPZ demyelination revealed that adoptive transfer of myelin-reactive T cells into CPZ-fed mice had significant immune cell infiltration and preservation of demyelinated axons, though these axons were reduced in diameter (Baxi et al., 2015). Other models using CPZ include inducing biochemical modifications to myelin to produce a secondary immune response, which resulted in myelin-reactive splenocytes and MS-like lesions (Caprariello et al., 2018). These models are important as they reflect a “neurodegeneration first” model in which brain-intrinsic pathology triggers the peripheral immune response.

## Opcs in Neuroinflammation and Neurodegeneration

A vital role for OPCs has been well-described with respect to remyelination in MS lesions and in several experimental models (Franklin and Ffrench-Constant, 2008; Chang et al., 2012; Staugaitis et al., 2012; Hughes et al., 2013; Young et al., 2013; Kremer et al., 2015). It has also recently been shown that heterogeneity exists between OLs during MS, which may contribute to disease progression (Jakel et al., 2019) or could be a result of OPC plasticity to repair damage (Foerster et al., 2019). However, there is also evidence of additional non-remyelinating functions of OPCs that may actively contribute to the development of neuroinflammation. Initial demyelinating insults stimulate recruitment of OPCs into early lesion sites and induce proliferation (Boyd et al., 2013; Takahashi et al., 2013). While this cellular expansion can lead to the replenishment of OLs through activation of regenerative genes such as *Olig2* and *Sox2* (Zhao et al., 2015; Tiane et al., 2019), a deficiency in the ability of OPCs to differentiate into OLs contributes to demyelination and axonal damage within lesions, leading to neurodegeneration (Chang et al., 2002; Boyd et al., 2013; Gruchot et al., 2019). Inflammation has been identified as a key factor in the suppression of OPC differentiation, as differential expression of surface receptors allows OPCs to have a very broad and diverse response to cytokine stimulation (Schmitz and Chew, 2008; Moore et al., 2015). While activation of inflammatory pathways in OPCs is associated with cytotoxicity as well as stunted differentiation and maturation (Moore et al., 2015), it also leaves them primed to modulate immune function.

**Figure 1:**
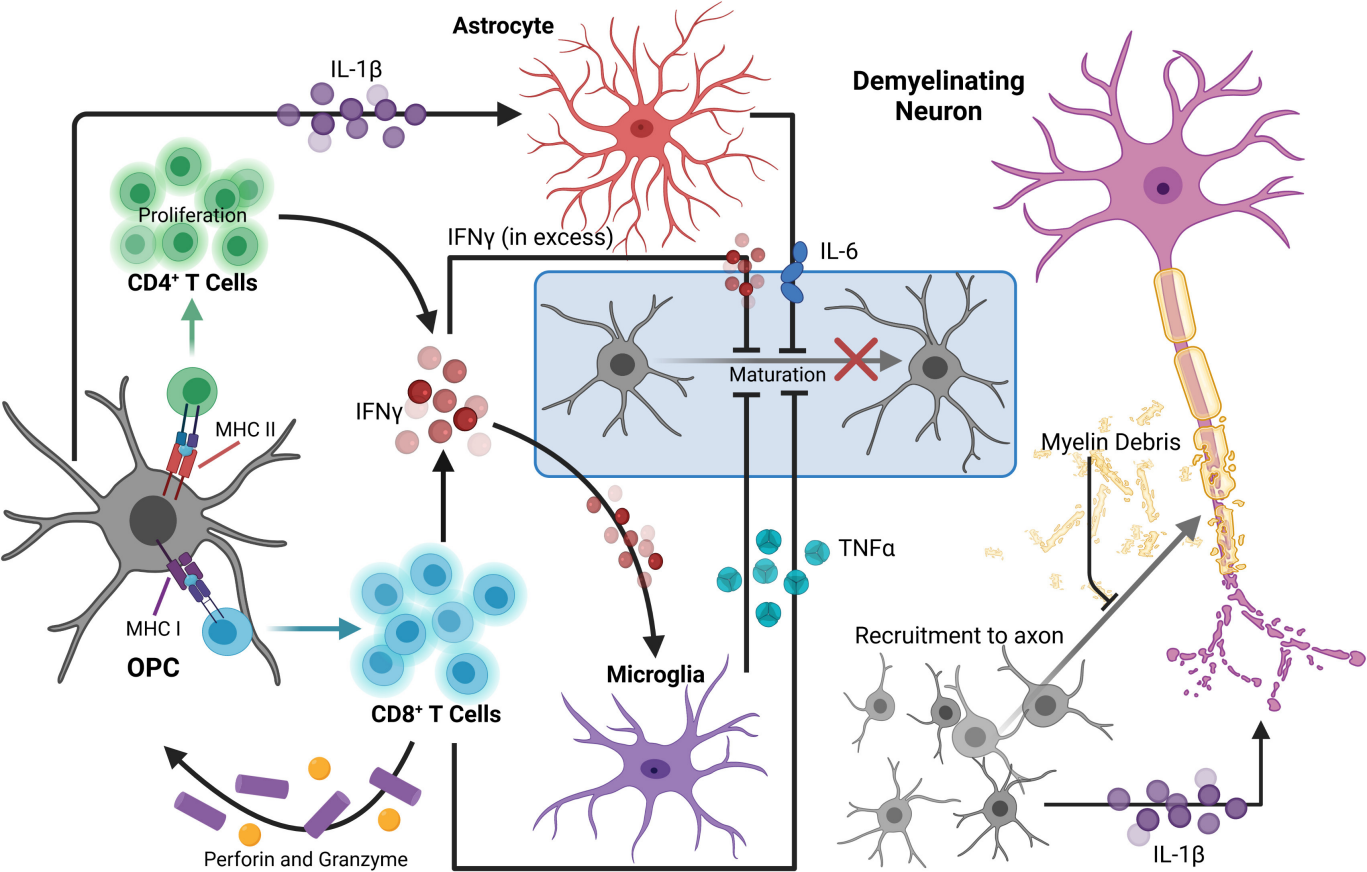
OPCs contribute to the perpetuation of inflammation, leading to enhanced neurodegeneration in MS. During neuroinflammation, IFNγ can upregulate both MHC I and II antigen presentation molecules on OPCs to promote functional antigen presentation to CD4^+^ and CD8^+^ T cells, respectively. The antigen-MHC I complex can then induce TNFα and IFNγ expression by CD8^+^ T cells, as well as perforin and granzyme secretion, which can contribute to localized OPC cell death. OPCs also promote proliferation of and cytokine production by CD4^+^ memory and effector T cells via MHC II antigen presentation. TNFα produced by microglia in response to IFNγ signaling by T lymphocytes can also impair OPC differentiation. Excess extracellular IFNγ is associated with inhibition of OPC maturation and oligodendrocyte cell death. OPC expression of IL-1β is also increased by IFNγ signaling, and stimulates IL-6 production by astrocytes, which can directly reduce OPC differentiation capacity. IL-1β produced by OPCs may also contribute to neuronal cytotoxicity directly by enhancing cytotoxic glutamatergic synaptic transmission. Myelin debris from degenerating myelin sheathes repel OPCs, inhibiting recruitment and maturation. Taken together, in a neuroinflammatory state, there are many barriers limiting axonal support, repair, and remyelination by OL lineage cells that are perpetuated by OPCs themselves. Created with BioRender.com.

Under homeostatic conditions, OPCs express low levels of inflammatory genes, which are normally seen in microglia, that are critical for environmental surveillance and movement to areas of demyelination (Fernandez-Castaneda and Gaultier, 2016; Voronova et al., 2017). In a disease state such as MS, expression of these genes can be significantly upregulated, potentially contributing to participation of OPCs in neuroinflammation, and further myelin and axon damage. Single-cell RNA sequencing of OPCs isolated from spinal cord tissue of mice with EAE revealed transcriptomic splicing that yielded upregulated disease-associated transcripts. Major histocompatibility (MHC) class I and II genes were highly expressed in EAE-derived OPCs compared to those in control mice, which was mediated by secretion of IFNγ by invading leukocytes (Figure 1). Furthermore, OPCs from EAE mice had increased phagocytic capacity as well as an ability to promote proliferation of and cytokine production from CD4^+^ memory and effector T cells via MHC II antigen presentation. Importantly, OPCs stimulated with IFNγ and/or MOG_35−55_ were not able to induce proliferation of naïve CD4^+^ T cells (Falcao et al., 2018), suggesting that priming of T cells first by professional antigen presenting cells is necessary for T cell interactions with antigen presented by OL lineage cells.

Similar relationships between OPCs and CD8^+^ T cells have been demonstrated using the CPZ model of demyelination. Adoptive transfer of myelin reactive T cells following CPZ treatment revealed that exposure to IFNγ upregulates MHC I antigen presentation machinery and immunoproteasome subunits in OPCs, promoting TNFα and IFNγ expression by CD8^+^ T cells. Perforin and granzyme secretion were also stimulated, promoting OPC-targeted cell death in the corpus callosum *in vivo* (Figure 1). Further, analysis of postmortem MS tissue revealed abundant expression of the immunoproteasome gene *PSMB8* in SOX10^+^ OL lineage cells specifically in demyelinated white matter lesions (Kirby et al., 2019). This suggests that immunoproteasome upregulation in OPCs may impair remyelination 2-fold: by active destruction of OPCs and shifting OPCs toward an immune-reactive state, contributing to chronic neurodegeneration observed in progressive MS patients (Basler et al., 2014; Kirby et al., 2019). Activation of immunomodulatory function in relation to antigen presentation by OPCs is also linked to extracellular receptors involved in phagocytosis and cytokine production, such as lipoprotein receptor-related protein 1. Further, specific deletion in OLs and OPCs led to a reduction of both infiltrating cells and MHC I expression by OPCs in EAE and in CPZ (Fernández-Castañeda et al., 2020). Together, these studies suggest that OPCs are active contributors to ongoing inflammation by direct stimulation of autoreactive T cells via myelin antigen presentation during MS and in several models of demyelination.

In addition to antigen presentation molecules, OPCs have also been shown to produce select inflammatory cytokines and chemokines in areas of demyelination. Gene expression analysis of OPCs from control and CPZ-treated mice demonstrated increased expression of IL-1β in activated PDGFRα^+^ OPCs from demyelinated regions (Moyon et al., 2015; Figure 1). IL-1β is a strong inducer of the innate immune response and has been detected within lesions and in the cerebrospinal fluid (CSF) of MS patients (Dujmovic et al., 2009; Burm et al., 2016). Further, OPCs were found to be a prominent producer of IL-17 during EAE as deletion of the upstream signaling molecule Act1 specifically in NG2^+^ cells drastically reduced EAE severity. Importantly, the same was not true for other CNS cells including neurons, mature OLs, and astrocytes (Kang et al., 2013). Of note, this reduction in EAE severity due to decreased OPC-derived IL-17 was likely not a direct effect on OPCs themselves since it was later found that IL-17A caused OPCs to exit the cell cycle and differentiate (Rodgers et al., 2015). In addition to the secretion of cytokines, chemokines involved in immune cell recruitment were also expressed by OPCs derived from the brains of mice treated with CPZ. These chemokines also included monocyte and microglia localizing cues like CCL2, which was significantly upregulated by Olig1^+^ cells within active, demyelinating lesions and along the borders of chronic lesions (Moyon et al., 2015). Taken together, the upregulation of genes associated with chemotaxis as well as modulation of inflammatory responses demonstrates a more direct role for OPCs in neuroinflammation through active participation in immune cell recruitment.

Another way OPCs are known to contribute to neuroinflammatory processes is through their dynamic interactions with the CNS vasculature. Active lesion analysis of MS patient tissue showed OPC clustering around vessels, indicating impaired perivascular migration. Grouping of OPCs at these sites was found to result from abnormal Wnt signaling that prevented detachment of OPCs from vessels. This was demonstrated in several *in vivo* models and caused improper astrocytic endfeet interactions with the vasculature and altered integrity of endothelial tight junctions via release of Wif1, which reduced claudin-5 expression. The inability of OPCs to detach from CNS vasculature led to a deficiency in OPC recruitment to demyelinated areas as well as disruption of the BBB (Niu et al., 2019). OPCs have also been shown to mediate opening of the BBB just prior to demyelination in white matter injuries through upregulation of matrix metalloproteinase (MMP)9 in response to IL-1β (Seo et al., 2013). MMP9 mediates proteolysis of the basal lamina and tight junction proteins, which can contribute to BBB permeability and ultimately result in an influx of peripheral immune cells, further contributing to demyelination and chronic neuroinflammation (Mirshafiey et al., 2014).

Sustained inflammatory activity by OPCs may also contribute to neurodegeneration as a deviation toward this phenotype contributes to a dysregulation in myelin production, which critically impacts axonal survival and proper axon conduction (Lubetzki and Stankoff, 2014; Seidl, 2014). Specifically, OPCs in an immune reactive state have a reduced ability to differentiate, reducing their capacity to remyelinate lesioned areas due to a decline in newly formed OLs. Cytokine production by OPCs, such as the production of IL-1β, may impact differentiation to OLs, as it is known to stimulate a number of cytotoxic cytokines from activated immune cells and glia, including IL-6 by astrocytes, which can inhibit OL maturation (Schonrock et al., 2000; Moore et al., 2015; Petkovic et al., 2016; Figure 1). TNFα produced by microglia in response to IFNγ signaling by T lymphocytes can also impair OPC maturation through processes such as mitochondrial dysfunction or cytotoxicity (Kim et al., 2011; Bonora et al., 2014). Likewise, excess extracellular IFNγ during acute inflammatory conditions has been associated with inhibition of OPC maturation and induction of OL cell death during MS (Vartanian et al., 1995; Falcao et al., 2018; Kirby et al., 2019; Figure 1). Furthermore, chronic MS lesions typically contain immature OPCs suggesting that OPCs are prevented from differentiating, rather than lost, and this may be a primary contributor to remyelination failure in some neurodegenerative diseases, including MS (Chang et al., 2002; Kuhlmann et al., 2008).

Finally, OPCs can contribute to neurodegeneration by directly or indirectly impacting neuron survival and function via cytokine signaling. IL-1β produced by OPCs may contribute to neuronal cytotoxicity as excessive IL-1β has been shown to enhance cytotoxic glutamatergic synaptic transmission, which results in p53-mediated apoptosis (Rossi et al., 2014a) (Figure 1). Additionally, stimulation of cytokine production from CD4^+^ and CD8^+^ T cells by OPC antigen presentation can negatively impact neuronal health. Notably, IFNγ has been heavily implicated in MS pathology, as it is known to promote Th1 responses (Olsson, 1992; Fletcher et al., 2010), but it may also impact neurons directly by enhancing glutamate toxicity through dysregulation of α-amino-3-hydroxy-5-methyl-4-isoxazolepropionic (AMPA) receptors, which results in calcium influx, nitric oxide (NO) production, and a decrease in mitochondrial function (Mizuno et al., 2008). Moreover, release of TNFα from T cells and other immune cells can also affect glutamatergic transmission in neurons by promoting sustained AMPA receptor activity. This sustained activity results in dendritic spine loss and alterations in synaptic activity, which also contributes to neurodegeneration (Centonze et al., 2009). As a result, the prevention of OPC immune reactivity presents an interesting avenue for the reduction of neuroinflammation and neurodegeneration, and may lead to novel treatment options for MS patients.

## OPC Influencers During Neuroinflammation and Neurodegeneration

Many of the factors that influence OPC function during neuroinflammation are downstream of the cytokines IFNγ, TNFα, and IL-1β (Table 1). While the inflammatory effects of these cytokines actively contribute to microglial-led degeneration (Brück et al., 1996; Pang et al., 2010; Domingues et al., 2016; Cignarella et al., 2020), these same factors are necessary not only for effective myelin debris clearance (Lampron et al., 2015), but also for providing many of the factors responsible for the migration of OPCs and fostering their maturation (Nicholas et al., 2002; Pasquini et al., 2011; Vogel et al., 2013). Many of these factors are secreted by other CNS resident cells including, but not exclusive to, microglia, astrocytes, and neurons (Table 1).

**Table 1:** CNS-derived factors that modulate OPC function.

**Factor**	**Type**	**Upstream Signal**	**Source**	**OPC Effect**	**Promote (+)/Inhibit (−)**	**References**
CCL2	Chemokine	IL-1β	Astrocytes	Migration	+	Glabinski et al., 1996; Wang et al., 2014; Moyon et al., 2015
CCL3	Chemokine	IFNγ	Astrocytes Microglia	Migration	–	Couturier et al., 2016; Shen et al., 2021
CCL11	Chemokine	IFNγTNFα	Astrocytes Microglia	MigrationProliferationMaturation	+/–	Maysami et al., 2006a; Ding et al., 2015; Parajuli et al., 2015
CXCL1	Chemokine	IL-1β	Astrocytes Microglia	MigrationProliferation	+	Robinson et al., 1998; Tsai et al., 2002; Omari et al., 2005; Karim et al., 2019; Michael et al., 2020
CXCL10	Chemokine	IFNγ	Astrocytes Microglia	Maturation	–	Luster et al., 1985; Vanguri and Farber, 1994; Xia et al., 2000; Tirotta et al., 2011; Moore et al., 2015
CXCL12	Chemokine	IL-1βTNFα	Astrocytes	MigrationProliferation Maturation	+	Maysami et al., 2006b; Patel et al., 2010; Cruz-Orengo et al., 2011; Luo et al., 2016
IFNγ	Cytokine	Other	Microglia	ProliferationMaturation	+/–	Chew et al., 2005; Lin et al., 2006; Tanner et al., 2011; Hindinger et al., 2012
IL-1β	Cytokine	Other	Astrocytes Microglia	MigrationProliferationMaturation	+/–	Vela et al., 2002; Wang et al., 2015; Zhou et al., 2017
IL-6	Cytokine	IL-1β	Astrocytes Microglia	Maturation	+/–	Schonrock et al., 2000; Moore et al., 2015; Petkovic et al., 2016
IL-11	Cytokine	IL-1β	Astrocytes	ProliferationMaturation	+	Zhang et al., 2006; Gurfein et al., 2009
LIF	Cytokine	IFNγ	Astrocytes	Maturation	+/–	Butzkueven et al., 2002; Stark et al., 2004; Ishibashi et al., 2006
TNFα	Cytokine	IFNγ	Microglia	Proliferation	+/–	Patel and Klein, 2011; Welser-Alves and Milner, 2013
Anosmin-1	ECM	Other	Astrocytes	Migration	–	Bribián et al., 2008
CSPGs (family)	ECM	IL-1β	Astrocytes	MigrationMaturation	–	Anderson et al., 2016; Sun et al., 2017b
Fibronectin	ECM	Other	Astrocytes Microglia	MigrationMaturation	+/–	Stoffels et al., 2013
Hyaluronan	ECM	Other	Astrocytes	Maturation	–	Back et al., 2005; Nair et al., 2008
Laminin	ECM	Other	Astrocytes	ProliferationMaturation	+	Chun et al., 2003
Tenascin-C	ECM	IFNγ	Astrocytes	MigrationProliferation	+/–	Garcion et al., 2001; Ogawa et al., 2005
Tenascin-R	ECM	Other	Astrocytes	Maturation	+	Czopka et al., 2009; Okuda et al., 2014
Vitronectin	ECM	Other	Astrocytes	Proliferation	+	Milner et al., 1999; Baron et al., 2002
BDNF	Growth Factor	TNFα	Astrocytes Microglia	ProliferationMaturation	+	Ferrini and De Koninck, 2013; Miyamoto et al., 2015
CNTF	Growth Factor	Other	Astrocytes	Maturation	+	Dallner et al., 2002; Talbott et al., 2007
FGF2	Growth Factor	IL-1β	Astrocytes Microglia	MigrationProliferation	+	Clemente et al., 2011
IGF-1	Growth Factor	IL-1βIFNγ	Astrocytes Microglia	ProliferationMaturation	+	Mason et al., 2001; Lin et al., 2005; Butovsky et al., 2006
IGF-2	Growth Factor	IFNγ	Microglia	Maturation	+	Nicholas et al., 2002
NT3	Growth Factor	Other	Astrocytes	Proliferation	+	Wong et al., 2013
PDGF-AA	Growth Factor	TNFα	Astrocytes	MigrationProliferation	–	Silberstein et al., 1996; Fruttiger et al., 1999; Baron et al., 2000, 2002; Frost et al., 2003
VEGFA	Growth Factor	IL-1βIFNγ	Astrocytes	Migration	+	Hayakawa et al., 2011; Argaw et al., 2012
Triiodothyronine	Hormone	Other	Astrocytes	Maturation	+	Dugas et al., 2012; Dezonne et al., 2013
Prostaglandin E_2_	Lipid	IL-1β	Astrocytes Microglia	Maturation	–	Marusic et al., 2005; Shiow et al., 2017
Adenosine	Neurotransmitter	Other	Neuron	ProliferationMaturation	+	Stevens et al., 2002; Safarzadeh et al., 2016
ATP	Neurotransmitter	IFNγ	Astrocytes Neurons	MigrationMaturation	+/–	Verderio and Matteoli, 2001; Agresti et al., 2005; Hamilton et al., 2010
GABA	Neurotransmitter	IL-1β	Neuron	Migration	+	Lin and Bergles, 2004; Tong et al., 2009
Glutamate	Neurotransmitter	IL-1β	Astrocytes Neuron	MigrationMaturation	+	Haberlandt et al., 2011; Zonouzi et al., 2011; Li et al., 2013; Xiao et al., 2013
BMP2, 4, 6, and 7	Protein	IFNγ	Astrocytes Neurons	Maturation	–	Ara et al., 2008; Miyagi et al., 2012
Galactin-3	Protein	Other	Astrocytes Microglia	Maturation	+	Pasquini et al., 2011; Itabashi et al., 2018; Thomas and Pasquini, 2018; Ramírez Hernández et al., 2020
Semaphorin 3A	Protein	Other	Astrocytes Microglia	Migration	–	Spassky et al., 2002; Syed et al., 2011; Yamaguchi et al., 2012
Semaphorin 3F	Protein	Other	Astrocytes Microglia	Migration	+	Spassky et al., 2002
Semaphorin 4D	Protein	Other	Astrocytes Microglia	Migration	–	Spassky et al., 2002; Yamaguchi et al., 2012
TIMP-1	Protein	IL-1β	Astrocytes	Maturation	+	Crocker et al., 2006; Welser-Alves et al., 2011; Nicaise et al., 2019b
Wnt (family)	Protein	IL-1βTNFα	Astrocytes Neurons	Maturation	+/–	Fancy et al., 2009; Feigenson et al., 2009; Edara et al., 2020; Guérit et al., 2021
Retinoic Acid	Morphogen	Other	Astrocytes	ProliferationMaturation	+	van Neerven et al., 2010; Shearer et al., 2012; Morrison et al., 2020

### Cytokines

IFNγ is known to drive acute autoimmune neuroinflammation by activating antigen presenting cells, promoting the differentiation of Th1 cells, and regulating peripheral immune cell infiltration into the CNS (Lees et al., 2008; Fletcher et al., 2010). Astrocytes treated with IFNγ can promote the proliferation and differentiation of myelin-specific Th1 cells (Dong and Benveniste, 2001) and upregulate localizing cues to direct CNS immune cell entry during acute EAE (Rosenman et al., 1995; Williams et al., 2020). While the most characterized effects of IFNγ during acute disease are primarily pathogenic, the role of IFNγ signaling during chronic stages of MS and in models of neuroinflammation, when the presence of the peripheral immune cells are reduced, remain incompletely understood. In fact, there is mounting evidence to suggest that IFNγ also has protective functions, particularly during chronic disease (Furlan et al., 2001; Sosa et al., 2015; Sun et al., 2017a; Smith et al., 2020). Further, some discrepancy exists regarding the effects of IFNγ on OPCs, likely due to differences in dosage and/or model system. For example, *in vitro*, low levels of IFNγ, while non-apoptoic, were demonstrated to inhibit OPC cell cycle exit, which bolstered the population of immature OPCs and reduced maturation into O1^+^ OLs (Chew et al., 2005). A later study also reported that even lower doses of IFNγ were not toxic to OPCs, but that IFNγ treatment led to a downregulation of the immature OPC marker platelet-derived growth factor receptor (PDGFR)α and the proliferative marker Ki-67, causing OPCs to reversibly exit the cell cycle (Tanner et al., 2011), which is necessary for maturation. Further, after sustained exposure to non-toxic, low-dose IFNγ, induced pluripotent stem cells from peripheral blood mononuclear cells from progressive MS patients differentiated into OL lineage cells had reduced differentiation (Morales Pantoja et al., 2020; Starost et al., 2020). This highlights the complexity of IFNγ signaling and need for further studies to elucidate its many roles during neuroinflammation and neurodegeneration.

TNFα is another pleiotropic cytokine with multiple disparate cellular responses including apoptosis, cell survival, and proliferation, and its function is receptor- and cell-context dependent (Locksley et al., 2001). In MS patients, TNFα expression was higher in lesions compared to non-inflammatory neurological disease controls and is detectible in the CSF (Cannella and Raine, 1995; Burman et al., 2014). However, following injection of TNFα into uninjured brains, OPCs did not upregulate NG2 expression, suggesting that exogenous TNFα alone does not activate OPCs *in vivo* (Rhodes et al., 2006). Although TNFα receptor (TNFR)1 is expressed by OPCs and is known to mediate neurotoxic functions (Su et al., 2011), TNFR2 signaling largely correlates with CNS repair and immune modulation either via activation in cells of the OL lineage themselves (Madsen et al., 2020) or in astrocytes or microglia, leading to downstream mediators of OL maturation and myelination (Patel et al., 2012; Fischer et al., 2014; Gao et al., 2017). Indeed, clinical trials using lenercept, an inhibitor of TNFR1 action, resulted in increased frequency, duration, and severity of MS relapses (1999).

IL-1 signaling has also been implicated in both MS pathogenesis and repair and has many roles within the CNS. It is thought to have many functions in the promotion of the inflammatory response as mice deficient in IL-1 receptors are protected from the development of neuroinflammation and EAE (Matsuki et al., 2006). Further, IL-1β in particular, is found to be highly concentrated in MS lesions, and is likewise increased in the CSF of MS patients (Cannella and Raine, 1995; Rossi et al., 2014b). Conversely, IL-1β was necessary for remyelination after CPZ-induced demyelination of the corpus callosum, as IL-1β-deficient animals had reduced IGF-1 production, delaying OPC maturation (Mason et al., 2001). Despite its role in promoting growth factor production necessary for CNS repair, like TNFα, IL-1β is thought to be a risk factor in MS (De Jong et al., 2002). Given the many targets of IFNγ, TNFα, and IL-1β, their prominent roles in cell-cell communication within the CNS, and variations in their expression over the course of acute and chronic neuroinflammatory disease, these cytokines remain relevant targets of MS research.

### Cell Types

#### Microglia

Microglia are considered the resident macrophage of the CNS. They originate from early yolk sac progenitors (Ginhoux et al., 2010; Kierdorf et al., 2013; Gomez Perdiguero et al., 2015) and are critical for proper CNS development, maintaining homeostasis, and mounting an inflammatory response in the event of a pathogenic insult (Butovsky and Weiner, 2018). Microglia are self-renewing via stable turnover throughout the lifespan and are maintained without the contribution of peripheral monocytes under homeostatic conditions (Askew et al., 2017). It is well-established that microglia heavily impact neuronal function, refining synapse connectivity and providing trophic support (Paolicelli et al., 2011; Schafer et al., 2012). They also have sophisticated communication networks with other glia including astrocytes and OPCs (Domingues et al., 2016; Linnerbauer et al., 2020). Like all glial subtypes, microglia exhibit a high degree of heterogeneity and plasticity (Bottcher et al., 2019; Masuda et al., 2020), are heavily influenced by the microenvironment, and can significantly contribute to neuroinflammation and CNS recovery (Gosselin et al., 2014; Lavin et al., 2014; Voet et al., 2019).

**Figure 2:**
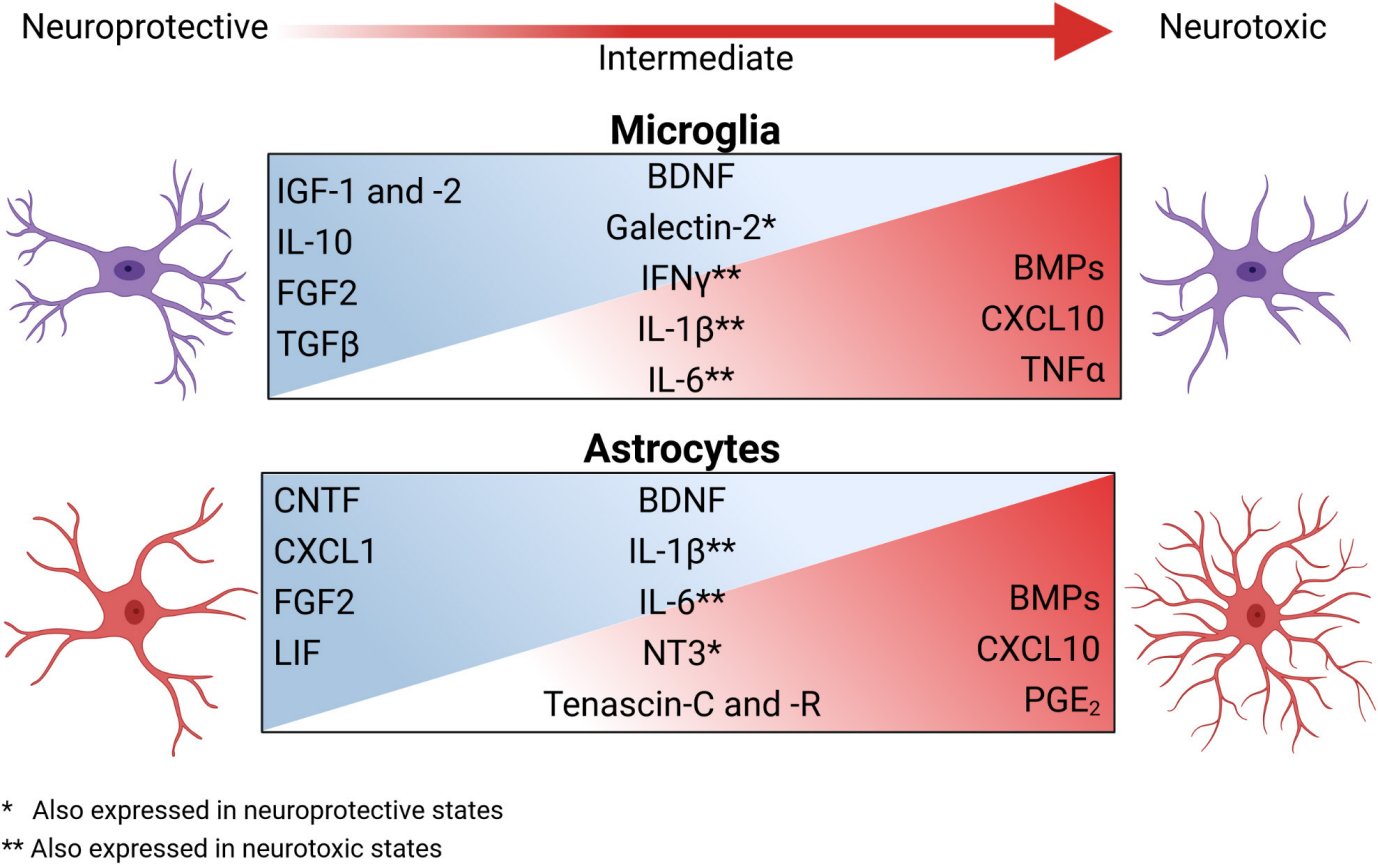
The secretory profiles of glia change across the spectrum of reactive phenotypes and have broad impacts on OPC function during neuroinflammation and neurodegeneration. During homeostasis, astrocytes and microglia interact with other CNS resident cells to help maintain a homeostatic environment via the secretion of trophic factors. During neuroinflammation, the microenvironment changes both cellularly and molecularly to shift the activation state of these glia, inducing a spectrum of glial secretory profiles. Factors secreted during this shift include growth factors, cytokines, survival signals, and ECM modulators. Classically activated microglia and astrocytes are highly inflammatory and significantly contribute to microenvironmental neurotoxicity and inhibit OPCs by secreting bone morphogenic proteins (BMPs), chemokines, cytokines, and prostaglandin E2 (PGE_2_). As inflammation resolves, the reactive state of microglia and astrocytes again promote the reparative properties of OPCs. Created with BioRender.com.

OPCs have differing responses to the various inflammatory states of microglia, partially due to a difference in trophic factors expressed by specific activation states (Pang et al., 2000, 2010; Domingues et al., 2016; Hagemeyer et al., 2017; Figure 2). Classically activated microglia are highly inflammatory and tend to inhibit OPC differentiation (Kigerl et al., 2009) as well as induce pro-apoptotic signals via excessive TNFα signaling. Comparatively, steady-state microglia aid OPCs primarily via the secretion of various trophic factors (Hanisch and Kettenmann, 2007; Hagemeyer et al., 2017), which are typically aimed at maintaining homeostasis and facilitating OPC proliferation (Table 1). As such, these quiescent microglia tend to secrete IGF-2 and galectin-2, which prompt OPC proliferation and prime OPCs for other differentiation signals (Nicholas et al., 2001, 2002; Pasquini et al., 2011; Hoyos et al., 2014; Figure 2). Likewise, alternatively activated microglia also express many of the same trophic factors secreted by both steady-state and highly inflammatory microglia, but in more moderate amounts (Domingues et al., 2016; Cignarella et al., 2020; Wang et al., 2020). These alternatively activated microglia express more galectin-2 than steady-state microglia, which further promote OPCs differentiation (Nicholas et al., 2002; Pasquini et al., 2011; Hoyos et al., 2014). Additionally, they produce moderate amounts of TNFα and NO, which did not exert cytotoxic effects and promoted OPC differentiation (Nicholas et al., 2002; De Jager et al., 2009; Karamita et al., 2017). Of note, while steady-state, classically activated, and alternatively activated microglia are well-studied, disease settings present a host of intermediate and unique activation states that lead to responses that are widely varied (Brück et al., 1996; Miron et al., 2013; Domingues et al., 2016; Wang et al., 2020).

In addition to the secretion of various inflammatory and/or tissue protective factors, microglia significantly alter the microenvironment through their phagocytic functions. During MS, microglia are the primary contributors to the clearance of myelin debris (Davalos et al., 2005; Lampron et al., 2015; Lloyd and Miron, 2019; Cignarella et al., 2020). Without myelin debris removal, proper remyelination by OPCs is significantly hindered, regardless of the remyelination-promoting factors present (Lampron et al., 2015; Cignarella et al., 2020; Figure 1). Myelin debris also contributes to inflammation and can activate microglia (Williams et al., 1994; Vogel et al., 2013). Within chronic-active lesions, the lesion edge houses a majority of the myelin debris, which promotes the formation of the microglial ring and enhances inflammatory activation (Frischer et al., 2015; Kuhlmann et al., 2017). Excessive activation of microglia can further damage OLs, contributing to the slowly expanding nature of chronic-active lesions (Dal-Bianco et al., 2017; Calvi et al., 2020). During active clearance, microglia also send inhibitory signals that block OPC migration and differentiation (Williams et al., 1994; Vogel et al., 2013; Lampron et al., 2015). As myelin debris is cleared, inflammation is typically reduced and OPCs lose the inhibitory signals from the highly activated microglia ringing the lesion (Williams et al., 1994; Vogel et al., 2013; Dal-Bianco et al., 2017; Calvi et al., 2020). However, at this point in neurodegeneration, neurons have largely died and the glial scar has begun forming (Brück et al., 1996; Voskuhl et al., 2009; Kuhlmann et al., 2017), creating new barriers for OPC differentiation.

#### Astrocytes

Astrocytes have numerous essential roles in nearly all aspects of CNS function and to meet the complex needs of their surroundings, astrocytes are highly heterogenous. During homeostatic conditions, astrocytes provide many neurotropic factors, promoting the survival and growth of surrounding neurons and glia (Escartin et al., 2021; Figure 2). However, we and others have shown that during inflammatory events, such as in MS and EAE, astrocytes are highly responsive to cytokines and inflammatory mediators (Zhang and Barres, 2010; Daniels et al., 2017; John Lin et al., 2017; Smith et al., 2020; Williams et al., 2020). This diverse astrocytic response is critical in healthy tissue preservation and reducing prolonged CNS exposure to cytotoxic mediators. Indeed, ablation of astrocytes following several types of CNS injury led to sustained inflammation, impaired BBB repair, and increased neurodegeneration (Bush et al., 1999; Faulkner et al., 2004; Gao et al., 2005; Sofroniew, 2005; Myer et al., 2006; Voskuhl et al., 2009; Arai and Lo, 2010; Hayakawa et al., 2014; Anderson et al., 2016; Liddelow and Barres, 2017).

Reactive astrocytes act in concert with other CNS cells to sustain the neuroinflammatory response necessary for the resolution of pathogenic threats to the CNS. As is the case with microglia, OPCs respond differently to varying activation states of astrocytes (Su et al., 2011; Voskuhl et al., 2019; Willis et al., 2020). As concluded recently, the binary division of reactive astrocytes into neurotoxic and neuroprotective is far too limited to capture the diverse astrocytic subsets (Escartin et al., 2021). Astrocytes can heavily influence OPC function via their effect on the microenvironment (Kiray et al., 2016) and by modulating the extracellular matrix (ECM) (Risau and Wolburg, 1990; Sixt et al., 2001). Traditionally, the ECM is described as a scaffolding on which OPCs traffic to reach demyelinated areas in need of repair (Hu et al., 2009). More recently, the ECM deposited by astrocytes was also shown to provide directional cues to OPCs and specific signals to initiate differentiation (Lau et al., 2013; Wang et al., 2017). Modulatory components of the ECM that can affect the ability of OPCs to migrate, proliferate, or differentiate include fibronectin, anosmin-1, laminin, hyaluronan, and several members of the chondroitin sulfate proteoglycan (CSPG) family, along with many other ECM modifiers (Benarroch, 2015; Table 1). Of note, inflammatory astrocytes tend to express the ECM proteins tenascin C and R, which inhibit migration of OPCs while simultaneously promoting OPC differentiation (Frik et al., 2018; Table 1). While it is thought that the timing and level of astrocyte activation significantly influences the migration and differentiation of OPCs via modulation of the ECM, the exact role of the differing astrocytic subtypes on modification of the ECM is complex and an area of active research, which was recently reviewed in detail (Ghorbani and Yong, 2021).

Astrocytes modify the microenvironment beyond modulating the ECM. The most neurotoxic astrocytes cause cellular damage (Liddelow et al., 2017); however, not only do more neuroprotective astrocytes promote a more trophic environment, recent evidence shows that intermediates between these subtypes can mediate a wider variety of beneficial effects (Teo and Bourne, 2018; Bhatia et al., 2019; Figure 2). A spectrum of reactive astrocyte states are implicated in OPC migration, proliferation, and differentiation (Nutma et al., 2020; Wheeler et al., 2020; Escartin et al., 2021) and produce some inflammatory factors, which are necessary for myelin debris removal and a return to a stable microenvironment (Erta et al., 2012; Traiffort et al., 2020). For example, leukemia inhibitor factor (LIF), fibroblast growth factor (FGF)2, and ciliary neurotropic factor (CNTF) are expressed by astrocytes that are categorized as non-reactive, neuroprotective, and/or in an intermediate reactive state (Aloisi et al., 1994; Müller et al., 2007; Delgado-Rivera et al., 2009; Figure 2). Further, astrocytes may contribute to a return to tissue homeostasis via IFNγ-mediated upregulation of the immunoproteasome to degrade oxidatively damaged proteins and clear oxygen radicals at sites of inflammation (Smith et al., 2020). As with ECM-modifying factors, the microenvironment established by astrocytes is a balance of timing and level of response (Ponath et al., 2018). One prime example of the breakdown of this balance is the formation of the glial scar. The glial scar forms in chronic lesions in response to severe damage and consists primarily of reactive astrocytes (Dal-Bianco et al., 2017; Kuhlmann et al., 2017; Adams and Gallo, 2018). Traditionally, the glial scar is viewed as a double-edged sword, in that it limits the initial damage but precludes regeneration as reviewed by Adams and Gallo (2018). However, other evidence suggests that the glial scar releases trophic factors responsible for neuronal growth and OPC proliferation, migration, and survival including brain-derived neurotrophic factor (BDNF) and neurotrophin (NT)3 (Anderson et al., 2016; Haindl et al., 2019; Figure 2; Table 1). However, while axons within glial scars in other models remyelinate (Bradbury and Burnside, 2019), those in MS lesions are less likely to, particularly in lesions surrounded by an inflammatory border (Dal-Bianco et al., 2017; Kuhlmann et al., 2017). These lesions that progress from chronic active to the inactive quiescent state have very little if any remyelination due to the inability of OPCs to migrate (Kornek et al., 2000; Li et al., 2016).

#### Neurons

Neuronal communication with OPCs via neurotransmitters is altered in MS and in MS models. Early studies suggested glutamate signaling may block OPC proliferation (Gallo et al., 1996; Yuan et al., 1998); however, using lysolecithin, it was later found that demyelinated axons formed functional glutamatergic synapses with OPCs that migrated from the subventricular zone. As OPCs matured into OLs, glutamatergic signaling was lost, indicating a potential role for glutamate in the migration of OPCs to lesioned areas (Etxeberria et al., 2010). Another neurotransmitter, gamma-aminobutyric acid (GABA), while excitatory during development, is the primary inhibitory neurotransmitter in the mammalian CNS. OPCs express the GABA-A receptor and endogenous GABA may also affect OPC migration by inhibiting AMPA receptor-mediated glutamatergic signaling (Lin and Bergles, 2004). Additionally, GABA promoted OPC migration in rat brain slice cultures (Tong et al., 2009) and neuronal GABA signaling is impaired in EAE, particularly in the striatum (Rossi et al., 2011), along with excessive glutamate activity (Centonze et al., 2009). There are several indications that neurotransmitters play an important role in the function of OPCs during neuroinflammation; however, the precise mechanisms that may enhance or prevent neurodegeneration are yet to be fully elucidated.

In response to neuronal action potentials, OPCs upregulate adenosine receptors, which are largely beneficial. Adenosine is known to inhibit OPC proliferation and stimulate cell cycle exit via A1 adenosine receptors (A_1_ARs), allowing OPCs to differentiate into myelinating OLs (Stevens et al., 2002). Activation of A_1_ARs on OPCs was later shown to also promote migration (Othman et al., 2003). During EAE, A_1_AR-deficient mice had worsened axonal injury, demyelination, as well as upregulation of pro-inflammatory factors by microglia/macrophages resulting in enhanced OL death (Tsutsui et al., 2004). Conversely, stimulation of A_2A_ receptors inhibits OPC maturation, which is in contrast to the oligodendrogenesis promoting effects of A_1A_Rs (Coppi et al., 2013). Since OPCs form synapses with neurons during development and are electrically active (Káradóttir et al., 2008), it is believed that restoration of normal electrical activity in neurons could aid in remyelination and recovery from demyelinating insults. A study using lysolecithin-induced lesions showed that demyelinated axons were still able to propagate action potentials, which promoted OPC differentiation and remyelination within the lesion. This corresponded to an activity-dependent increase in the OPC pool in mice that received repeated neuronal electrical stimulation (Ortiz et al., 2019). While there is dysregulation of neuron-OPC crosstalk during ongoing neuroinflammation, the primary source of the secreted OPC-modulating factors are lymphocytes, microglia/macrophages, and astrocytes. Although neurons rely on myelin and mature OL support for proper functioning, they are nonetheless an important contributor to neuroinflammatory pathology and are critical to OPC-mediated CNS repair.

## Effects of Aging on OPCs

Aging is a significant risk factor for most neurodegenerative disorders (Minden et al., 2004; Hou et al., 2019), and is correlated with a more progressive disease course in patients diagnosed with MS over the age of 40 (Scalfari et al., 2011). With advancing age, MS disease progression tends to shift toward a less inflammatory, neurodegenerative state, as only one-third of patients continue with RRMS past the age of 75 (Tutuncu et al., 2013; Scalfari et al., 2014). The potential for clinical recovery is reduced with age and can ultimately contribute to neurodegeneration (Sanai et al., 2016). Specifically, as both patient age and disease duration increase, demyelination becomes more infrequent due to a decrease in acute inflammatory events; however, the capacity of OPCs to remyelinate damaged neurons also becomes impaired (Bramow et al., 2010; Ruckh et al., 2012). Previously demyelinated axons risk long-term exposure to chronic inflammation and can become irreversibly damaged as a result (Kornek et al., 2000).

Age-related remyelination failure has been linked to suppression of OPC differentiation as large pools of undifferentiated OL lineage cells were present in chronically demyelinated MS lesions in post-mortem tissue (Boyd et al., 2013). It has also been shown that functionally, OPCs become regionally heterogenous with age, suggesting that particular areas of the CNS may be at greater risk for long-term damage (Spitzer et al., 2019). Rat OPC cultures harvested from young (2–3 months) and aged (20–24 months) cohorts *in vitro* showed that the aged OPCs had a reduced capacity for differentiation. They also lacked proliferative growth factors, as roughly 20% expressed mature OL markers compared to 60% by OPCs from younger animals. Moreover, treatment of aged OPCs with maturation stimulating factors failed to increase the population of mature OLs efficiently, requiring weeks of additional time to fully mature compared to young OPCs, suggesting that aging induces intrinsic changes within OPCs that restrict their response to extracellular differentiation signals (Neumann et al., 2019). In another study, *in situ* hybridization analysis of OPCs in young (8–10 weeks) and aged (>1 year) rats undergoing EtBr-mediated demyelination revealed that OPCs in aged animals were recruited to the lesion site more slowly. Transcript levels of the OPC markers *Pdgfra* and *MyT1* were comparatively retained in aged rats and mature OL markers of *Mbp* and *Gtx* were delayed, indicative of impaired differentiation (Sim et al., 2002).

The intrinsic changes associated with both OPC differentiation and recruitment may be linked to cellular senescence, or the cessation of the cell cycle and a deviation from normal cell function toward an inflammatory state (Gorgoulis et al., 2019). Considered a hallmark of normal aging, cellular senescence is induced by a variety of stress signals related to DNA damage such as oxidative stress and release of inflammatory cytokines, all of which are associated with MS pathology (Haider et al., 2011; Elkjaer et al., 2019). In addition, cells that are senescent maintain the ability to release these factors, promoting cellular senescence in neighboring cells through paracrine signaling and generating pools of senescent cells that accumulate over time (Dimri et al., 1995; Ressler et al., 2006; Acosta et al., 2013). Aged OPCs have elevated levels of DNA damage as well as increased mRNA transcript levels of cellular senescence markers including *Cdkn2a* (Neumann et al., 2019). Upregulation of additional markers, such as senescence-associated beta-galactosidase, on SOX2^+^ OPCs resulted in inhibited differentiation suggesting that functional inhibition of OPC recruitment and differentiation may be partially explained by a shift toward a senescent phenotype (Nicaise et al., 2019a). Cellular senescence is not confined to cells with stem-like properties; it has also been documented in glial cells (Kritsilis et al., 2018), and may further affect the capacity of OPCs to mature and facilitate repair of damaged axons. Specifically, transcript analysis of astrocytes from young and aged rats showed an upregulation of genes associated with both senescence and inflammation such as *Mmp3, p53*, and *p21* in older astrocytes compared to those from younger rats. Similarly, there was a decrease in aged cells undergoing the G2 and S phases of the cell cycle, which was accompanied by an increase in cells associated with the G1 phase, demonstrating inhibited astrocytic proliferation (Willis et al., 2020).

Astrocytes are crucial for maintaining CNS microenvironments conducive to homeostasis and proper OPC function (Liedtke et al., 1996; Gadea et al., 2009; Kiray et al., 2016). One way astrocytes regulate the microenvironment is through release of extracellular vesicles. These vesicles are not exempt from age-related cellular effects, as vesicles purified from aged astrocytes had a reduced ability to support OPC differentiation (Willis et al., 2020), suggesting that age-induced senescence of other CNS resident cells may contribute to deficient remyelination in chronic MS lesions and can affect the remyelinating capacity of OPCs specifically. Another critical extrinsic microenvironmental cue that modulates OPC function is the mechanical stiffness of the progenitor niche. In an elegant study by Segel et al., interrogation of the OPC microenvironment revealed increased stiffness with age, which caused a loss of function of OPCs. This was reversed using scaffolds to mimic the “young” microenvironment, suggesting tissue stiffness is a critical regulator of aged OPCs (Segel et al., 2019a,b). Notably, apelin-APJ signaling in OL lineage cells increases the capacity for maturation, and a decrease in signaling has been implicated as a key factor in age-related remyelination impairment. Recently, activity of the APJ pathway has been shown to be significantly downregulated with age, as the primary ligand, apelin, is decreased in the plasma of older mice and correlates with APJ deficiency-induced hypomyelination in neonatal mice during development. Furthermore, mice induced with EAE as well as with toxin-mediated demyelination showed increased remyelination with activation of the APJ pathway *in vivo*, while also promoting transcript signatures of mature OLs in human OPCs (Ito et al., 2021). Stimulation of pro-differentiation pathways may be a viable treatment option to reverse the effects of aging on remyelination capacity and assist in reducing neurodegeneration in progressive stages of MS.

## Concluding Remarks

While it is clear that resident CNS cells contribute to ongoing inflammation during MS, the complexity of the signaling events in various cells types, the interactions between those cells, and the role these processes have in neurodegeneration are only beginning to be understood. This intricate cellular and molecular symphony can also vary widely depending on the subtype of MS, the stage of disease, and the age of the patient. Since remyelination failure is a significant roadblock in the treatment of MS (Franklin, 2002; Gruchot et al., 2019; Galloway et al., 2020), many early studies have focused on promoting OPC maturation to promote remyelination and prevent axon degeneration. Additionally, using multiple animal models of MS that recapitulate several aspects of the human disease, OPCs were shown to not only prevent neurodegeneration by participating in remyelination, but disease-specific OPCs were also identified and have the ability to present antigen to T cells, exacerbating neuroinflammation (Falcao et al., 2018; Kirby et al., 2019; Figure 1). Therefore, while elucidating the obstacles impeding remyelination is certainly critical to improve treatment options for MS patients and initiate recovery, more recent evidence suggests that OPCs have a much more versatile role in neuroinflammation and neurodegeneration, offering additional OPC-specific therapeutic targets.

In addition to factors intrinsic to OPC function during neuroinflammation and neurodegeneration in MS, there are a multitude of factors that impact the MS lesion microenvironment that critically regulate OPC migration, proliferation, and maturation (Figure 2; Table 1). Primary contributors to the inflammatory milieu are cytokines, which can be secreted from infiltrating peripheral leukocytes as well as other glia including microglia and astrocytes. Inflammatory cytokines like IFNγ, TNFα, and IL-1β are well-described in the literature as perpetuating inflammation by activation of infiltrated immune cells and surrounding glia and inducing cell death and damage to OLs, leading to demyelination and eventual neurodegeneration, particularly in early disease phases like RRMS. However, depending on the concentration, the responding cell type, and the receptors through which they signal, these same cytokines can have a variety of neuroprotective effects. Further, the activation state of neighboring microglia and astrocytes can strongly influence how OPCs respond to demyelinating insults by altering the ECM or the profile of trophic factors that they secrete (Figure 2), positioning OPCs at the center of many neuroinflammatory and neurodegenerative processes.

## Author Contributions

JW generated the concept design. MP, BS, RT, and JW wrote and edited this manuscript. MP created the figures. MP, BS, and RT created the table. All authors contributed to the article and approved the submitted version.

## Conflict of Interest

The authors declare that the research was conducted in the absence of any commercial or financial relationships that could be construed as a potential conflict of interest.
